# Training future endoscopists: gastroenterology fellows' perspectives and hands-on exposure to artificial intelligence for polyp detection in the United States

**DOI:** 10.1016/j.igie.2026.01.016

**Published:** 2026-02-02

**Authors:** Tessa Herman, Jason A. Dominitz, Tonya Kaltenbach, Andrew Gawron, Brian Hanson, Daniela Guerrero Vinsard

**Affiliations:** 1 Department of Gastroenterology, Hepatology, and Nutrition, Vanderbilt University Medical Center, Nashville, Tennessee, USA; 2 Gastroenterology Section, VA Puget Sound Health Care System, Seattle, Washington, USA; 3 Division of Gastroenterology, University of Washington, Seattle, Washington, USA; 4 Gastroenterology Section, San Francisco VA Health Care System, San Francisco, California, USA; 5 Division of Gastroenterology, University of California San Francisco, San Francisco, California, USA; 6 Gastroenterology Section, VA Salt Lake City Health Care System, Salt Lake City, Utah, USA; 7 Division of Gastroenterology, Hepatology, and Nutrition, University of Utah, Salt Lake City, Utah, USA; 8 Gastroenterology Section, Minneapolis Veteran Affairs Medical Center, Minneapolis, Minnesota, USA; 9 Department of Medicine, Division of Gastroenterology, Hepatology, and Nutrition, University of Minnesota, Minneapolis, Minnesota, USA

## Abstract

**Background and Aims:**

Artificial intelligence (AI)-assisted colonoscopy for polyp detection is designed to improve colonoscopy quality. Although surveys have assessed staff gastroenterologists' attitudes toward AI, less is known about the views of gastroenterology (GE) fellows regarding AI during training.

**Methods:**

We conducted a nationwide survey of GE fellows from August 2024 to November 2024 to assess (1) exposure to and experience with AI in fellowship, (2) perceptions of AI's impact on colonoscopy quality, and (3) attitudes toward implementing AI into training. The survey included Likert scale questions with branching logic to tailor questions based on AI availability at the fellows' institutions.

**Results:**

A total of 126 fellows started the survey, and 88 (69.8%) completed it. AI was available at least at 1 training site for 69.3% of respondents. In addition, 81.8% of fellows believed AI should be available during fellowship. Many fellows (43.2%) thought AI should be incorporated in the second year of training. Most fellows (60.7%) believed early exposure to AI-enhanced polyp detection skills. However, 52.5% felt neutral that AI made them better endoscopists overall. Despite this, 62.5% preferred to pursue a job with AI if they had trained with it.

**Conclusions:**

Our nationwide survey found that GE fellows are generally supportive of integrating AI into their training, with most advocating for its incorporation in the second year. These results should be considered by fellowship program leadership and GE practices recruiting fellows trained with AI. Further studies are required to assess the impact of training GE fellows with AI on their polyp detection competency.

## Introduction

Artificial intelligence (AI)-assisted colonoscopy with computer-aided detection (CADe) is a promising tool to improve the quality of colonoscopy, predominantly by increasing the number of adenomas detected during the procedure, as supported by recent systematic reviews and meta-analyses.^1^^,^^2^ Although its long-term impact on patient outcomes is uncertain, CADe has become increasingly common in gastroenterology (GE) practices over the past few years, and therefore, more GE fellows have been exposed to this technology.^3^

Prior studies exploring gastroenterologists' adoption of CADe have been largely aimed at staff gastroenterologists rather than GE trainees.^4^, ^5^, ^6^, ^7^ There are currently limited reported data regarding GE fellows' exposure to and experience with CADe, with most studies reporting fellows' perspectives on AI use without real hands-on exposure to it.^8^^,^^9^ Trainees’ exposure to AI during fellowship may influence their perspectives on AI use in their future practice, as well as their readiness to use AI in their career. We performed a nationwide survey aimed at assessing GE fellows' exposure to and attitudes toward implementation of CADe during their training and beyond. This survey informs the status of AI adoption during colonoscopy training in the United States from the trainee perspective.

## Materials and Methods

### Study design and aim

We conducted a nationwide, cross-sectional observational survey of GE fellows across the United States from August to November 2024. The aims of this study were to assess (1) exposure to and experience with CADe during GE fellowship, (2) perceptions of the impact of CADe on colonoscopy quality, and (3) attitudes toward implementing CADe into GE fellowship training.

### Study participants and recruitment

Our study targeted current GE fellows at any level of training and advanced fellows, with or without prior exposure to CADe. Fellows from university-based and community-based programs were invited to participate. Participants were recruited through several methods: we reached out to program directors and coordinators of all 115 university-based GE programs (according to the American Medical Association Fellowship and Residency Electronic Interactive Database Access) via e-mail and requested they forward the study survey to their fellows; we directly contacted fellows on social media (ie, X/Twitter); and we shared our survey QR code in-person via flyers at the American College of Gastroenterology (ACG) Annual Meeting 2024 in Philadelphia, Pa, USA. Participation in the study was voluntary, and no compensation was offered to participants in the study.

### Questionnaire

The 29-item anonymous survey was divided into 3 sections: (1) CADe exposure during fellowship, (2) attitudes toward and beliefs about CADe, and (3) demographic information (Supplementary Table 1, available online at www.igiejournal.org). The survey used branching logic to tailor questions based on prior CADe exposure. Thus, if fellows had not used CADe in their training, then the survey skipped ahead to only show questions that were relevant to attitudes and preferences about CADe for colonoscopy (ie, fellows were not asked questions about real-life experience with CADe). Branching logic was used to mitigate potentially irrelevant questions for respondents and mitigate survey fatigue. Questions were multiple choice, primarily using a 5-point Likert scale. Response to each question was optional. The Qualtrics survey tool (Qualtrics, Provo, Utah, USA) was used to create and send the survey. The survey underwent multiple rounds of development and refinement, incorporating feedback from 2 key groups: the study coauthors and pilot testing. All coauthors reviewed and provided input to the survey to ensure appropriate content and construct validity, logical flow, branching logic, consistency, and clarity. The survey was then piloted among GI fellows from the University of Minnesota program, which was the home institution of the first author (T.H.) and the principal investigator (D.G.V.).

### Statistical analysis

Descriptive statistics were used to summarize the findings of the study, presented as proportions for categorical variables or as a mean with standard deviation for numerical values. We further analyzed responses based on whether the fellow had real-life exposure to CADe.

### Institutional Review Board statement

The Minneapolis Veteran Affairs (VA) Health Care System Human Research Protection Program determined that this survey-based study did not meet the federal definition of research (Department of Health and Human Services 45 Code of Federal Regulations 46.102) and thus did not require Institutional Review Board oversight.

## Results

### Study population

**Figure 1:**
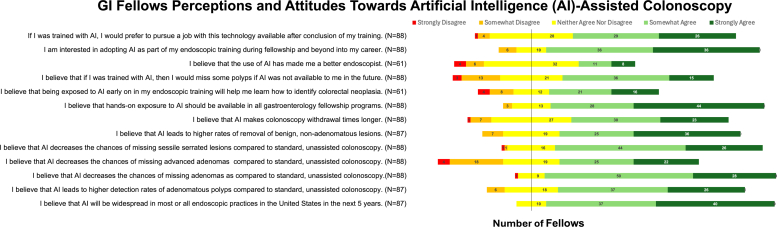
Gastroenterology (GE) fellows’ perceptions and attitudes toward artificial intelligence (AI)-assisted colonoscopy: survey study participants’ responses to statements regarding their perceptions and attitudes toward AI-assisted colonoscopy.

A total of 126 fellows started the survey, of whom 88 (69.8%) completed the survey and were included in the analysis. Fellows from all training years participated and represented various types of training sites (Table 1). Fellows performed a median of 200 colonoscopies (interquartile range, 50-500), including both CADe and non-CADe cases, by the time they replied to the survey. All survey responses are found in Figure 1 and Supplementary Table 1.

**Table 1:** Demographics of survey respondents and major findings

Demographics	
Characteristic	% (n)
Gender (n = 86)	
Woman	52.3% (45)
Man	45.3% (39)
Prefer not to answer	2.3% (2)
Current training year (n = 86)	
First-year fellow	25.6% (22)
Second-year fellow	33.7% (29)
Third-year fellow	34.9% (30)
Advanced fellow	4.7% (4)
Prefer not to answer	2.3% (2)
Fellowship program type (n = 88)	
University-based program	68.2% (60)
Community-based program	5.7% (5)
Did not respond	26.1% (23)
Practice plans after fellowship (n = 85)	
General gastroenterology	44.7% (38)
Hepatology	11.8% (10)
Inflammatory bowel disease	9.4% (8)
Advanced endoscopy	20% (17)
To be determined	14.1% (12)

Major findings	

Proportion of fellows with hands-on exposure to CADe	

Hands-on exposure to CADe	% (n)

AI is available for fellows to use	69.3% (61)
AI is available, but not available for fellow use	2.3% (2)
AI is not available	28.4% (25)

Year in fellowship CADe should be incorporated (n = 88)	

Year in training	% (n)

First year	31.8% (28)
Second year	43.2% (38)
Third year	18.2% (16)
Should not be incorporated	6.8% (6)

CADe availability at training sites∗	

Site	% (n)

University hospital (n = 58)	
CADe available to fellows	39.7% (23)
No limitations to use	82.6% (19)
Limited to second-year fellows and above	17.4% (4)
CADe restricted to attendings	6.9% (4)
CADe unavailable at site	53.4% (31)
Community hospital (n = 25)	
CADe available to fellows	24.0% (6)
No limitations	100% (6)
CADe restricted to attendings	0.0% (0)
CADe unavailable at site	76.0% (19)
Veteran Affairs Medical Center (n = 47)	
CADe available to fellows	97.9% (46)
No limitations to use	95.6% (44)
Limited to second-year fellows and above	2.2% (1)
Limited to third-year fellows and above	2.2% (1)
CADe restricted to attendings	2.1% (1)
CADe unavailable at site	0.0% (0)
Ambulatory surgery center (n = 15)	
CADe available to fellows	40.0% (6)
No limitations to use	66.7% (4)
Limited to second-year fellows and above	33.3% (2)
CADe restricted to attendings	6.7% (1)
CADe unavailable at site	53.3% (8)
Other (n = 1)	
CADe available to fellows	100% (1)
No limitations to use	100% (1)
CADe restricted to attendings	0.0% (0)
CADe unavailable at site	0.0% (0)

Colonoscopies completed using CADe	

No. of colonoscopies	% (n)

1-25	21.3% (13)
25-50	24.6% (15)
51-75	19.7% (12)
76-100	11.5% (7)
>100	23% (14)

*AI*, Artificial intelligence; *CADe*, computer-aided detection.

∗ These responses are based on the 61 fellows who stated that they have CADe available in at least 1 of their training sites.

### Exposure to CADe

Regarding exposure to CADe, the technology was available for fellows to use in at least 1 training site for 69.3% of respondents. CADe was most available to fellows at VA hospitals, followed by university hospitals, ambulatory surgical centers, community hospitals, and “other” sites (Table 1). Generally, fellows were not restricted from using CADe at most sites when it was available, although some sites limited CADe use to attendings only or second-year fellows and above (Table 1).

Fellows had varying experience with the quantity of colonoscopies completed using CADe. Fellows with exposure to CADe noted some flaws in the technology. For example, 66% (35/53) of fellows with CADe experience reported that they had visualized a polyp during a colonoscopy that was not detected or captured effectively with CADe, and 55.7% (34/61) of fellows reported that this happened often or very often.

Most fellows (79.4%, 50/63) agreed or somewhat agreed that the attendings they worked with were generally supportive of AI use in colonoscopy; only 2 fellows somewhat disagreed with this statement. Attendings generally permitted fellows to decide when to activate CADe during colonoscopies (always: 31.6% [18/57], most of the time: 28.1% [16/57], about half of the time: 8.8% [5/57], less than half of the time: 8.8% [5/57], and never: 21.1%[12/57]).

In terms of educational forums provided by their fellowship programs regarding the principles and application of CADe, fellows learned about it during lectures (63.9%, 23/36), journal clubs (44.4%, 16/36), simulations/workshops (11.1%, 4/36), or other educational means (8.3%, 3/36).

### Perceptions about CADe and its impact on colonoscopy quality

Respondents generally had positive perceptions of CADe. Most respondents (88.5%, 77/87) agreed or somewhat agreed that CADe will be widespread in most or all endoscopic practices in the United States in the next 5 years.

Respondents reported that CADe improved the quality of colonoscopy in terms of higher adenoma detection rates (72.4% [63/87] agreed or somewhat agreed), lower adenoma miss rates (88.6% [78/88] agreed or somewhat agreed), lower advanced adenoma miss rates (53.4% [47/88] agreed or somewhat agreed), and improved detection of sessile serrated lesions (79.5% [70/88] agreed or somewhat agreed). Conversely, fellows believed that CADe led to higher resection rates of benign, nonadenomatous lesions (70.1% [61/87] agreed or somewhat agreed) and longer withdrawal times (60.2% [53/88] agreed or somewhat agreed; Fig. 1). For the respondents who felt CADe led to longer withdrawal times, 92.5% (49/53) believed that this was because the endoscopist spent more time double-checking false-positive boxes, 45.3% (24/53) believed this was because CADe led to higher detection, and thus resection, of polyps, and 41.5% (22/53) thought this was because AI led the endoscopist to do a more thorough mucosal evaluation.

### Attitudes toward CADe implementation into GE fellowship training and beyond

Regarding attitudes toward CADe implementation during fellowship training, most respondents (81.8%, 72/88) agreed or somewhat agreed that hands-on exposure to CADe should be available in all GE fellowship programs. Many respondents (43.2%, 38/88) believed that it should be implemented during the second year of fellowship, as opposed to the first year (31.8%, 28/88) or third year (18.2%, 16/88) (Table 1). Fellows seldom (6.8%, 6/88) believed that CADe should not be implemented into fellowship training. Most fellows (62.5%, 55/88) agreed or somewhat agreed that if they were trained using CADe during fellowship, then they would prefer to pursue a job with CADe available (Fig. 1).

Most fellows (60.7%, 37/61) exposed to CADe in fellowship agreed that early exposure in their endoscopic training helps them learn to identify colorectal polyps. In addition, 58% (51/88) agreed or somewhat agreed that if they were trained with CADe, then they would miss polyps if CADe were removed from their practice. Lastly, 16.4% (10/61) of fellows who trained with CADe disagreed or somewhat disagreed that CADe made them a better endoscopist, and 52.5% (32/61) were neutral about this statement (Fig. 1).

## Discussion

In this nationwide survey targeting GE fellows to understand their real-world exposure and attitudes toward CADe, we found that many trainees are already using CADe during colonoscopy training. Overall, respondents expressed a positive outlook on its integration into fellowship and future endoscopic careers while also voicing concerns about its impact on training and skill development.

Nearly 82% of GE fellows in this study agreed that hands-on exposure to CADe should be available in all GE fellowship programs. Fellows believed that it improves colonoscopy quality and can help teach fellows to identify polyps, which has been supported by multiple single-center randomized controlled trials.^10^, ^11^, ^12^ In our study and in another single-center survey study (n = 10) by Magahis et al^9^ specifically targeting GE fellows, most trainees believed that CADe should be implemented during the second year of GE fellowship or later. This may reflect the belief that fellows must learn the foundations of endoscopic practice before implementing this technology. To determine the optimal timing for integrating CADe into endoscopic training, future research should include larger multicenter studies that objectively assess the impact of AI on the development of polyp detection skills. Complementary qualitative research—such as mixed-methods approaches, focus group discussions, and in-depth interviews with fellows—will be essential to capture nuanced perspectives and inform evidence-based curriculum design.

Other survey studies also found optimism regarding CADe and AI, more broadly, in GE. A cross-sectional study conducted by the American Society for Gastrointestinal Endoscopy AI Task Force found that nearly 96% of the 374 participants (including 62 GE fellows) believed that AI would positively impact the field of GE. However, in this survey, only 25 respondents had real-life exposure to CADe.^5^ Another study surveying ACG members followed a similar pattern of general support but minimal exposure to AI in practice.^8^ Similarly, in a nationwide survey study by Wadhwa et al^6^ that included 115 staff gastroenterologists and 6 GE fellows, 85.5% were interested in CADe technology. Magahis et al^9^ found that 9 of 10 respondents were interested in using AI for polyp detection in the future. This optimism is seemingly rooted in the belief that CADe will improve adenoma and sessile serrated lesion detection rates.^6^^,^^9^ This theory is supported by recent systematic review and meta-analyses showing that AI may improve adenoma and sessile serrated lesion detection rates.^2^^,^^13^ However, it is important to note that despite this, the American Gastroenterological Association Living Clinical Practice Guideline on CADe does not recommend or disapprove of its use after weighing its risks and benefits.^3^

We also identified negative perceptions about CADe implementation. Sixty percent of fellows believed that CADe leads to longer colonoscopy withdrawal times. This concern was recognized in prior studies among both staff gastroenterologists and GE fellows.^6^^,^^9^ In our study, the principal reason behind the perception of longer withdrawal times was that the endoscopist spends more time double-checking potential false-positive boxes, although some of the additional withdrawal time was attributed to increased detection of neoplasia. Similarly, most fellows (9/10) in the Mahagis et al^9^ study believed that AI would increase false-positive detections.^9^ Conversely, only 33.9% of gastroenterologists held this belief in Wadhwa et al's^6^ study. This may highlight a potential difference in perception of CADe performance between staff gastroenterologists and GE fellows. It can be difficult to realistically quantify the number of false-positive boxes, but current literature suggests that AI has trivial to no impact on withdrawal time.^2^^,^^3^^,^^13^

Another notable concern identified in our study was the concept of overreliance on CADe, in which the endoscopist may depend too heavily on the device to detect polyps and, thus, may not perform as careful an inspection (either consciously or subconsciously). Most fellows in our study believed that if they trained with CADe, then they would miss some polyps if CADe were not available to them in the future. Potential reasons for this may be fellows' fear to over-rely on AI for polyp detection versus the belief that AI makes them a better endoscopist and polyp detector. This concern has similarly been shared in other survey studies among GE fellows and staff alike.^6^^,^^9^ Multiple observational studies yielded mixed results on whether this deskilling occurs after CADe use, although there should be further exploration specifically evaluating trainees.^14^, ^15^, ^16^, ^17^ Notably, we did not measure whether and how trainees learned from CADe (eg, if they primarily looked for boxes to highlight polyps vs learned what a polyp looks like from CADe), so future studies should evaluate this.

Whether and how fellows are exposed to new technologies, such as AI, may significantly affect future practice patterns in GE. Trainee competency studies in the advanced endoscopy space have demonstrated that variability in the case volume, duration of training, and access to novel technologies or techniques results in unequal acquisition of skills among GE fellows.^18^^,^^19^ Those with AI exposure may be more comfortable and competent using it in their future practice. High-quality studies measuring the impact of CADe on endoscopic cognitive skills during training are needed to guide program directors and endoscopy educators. Programs currently using AI for colonoscopy may consider implementation of an AI curriculum, including didactic sessions covering the principles, capabilities, and limitations of AI technologies; simulated CADe use before using it on real patients; gradual exposure to CADe throughout fellowship (eg, the trainee starts without CADe but uses it more often as their foundational endoscopic skills develop); and clear benchmarks for manual polyp detection skills before introducing AI tools, ensuring that trainees develop core competencies independently of technology.

Our study has several limitations. First, we do not know definitively how many fellows received our survey, which is a limitation of the method of survey distribution, which we used in an attempt to get the highest response rate possible. Of those who did respond, we noted a significant dropout rate of fellows who started the survey but did not complete it (n = 38). Some reasons for this may include survey fatigue, the length of the survey, or the respondent was interrupted and unable to complete it. Similarly, fellows who are more interested in AI may have been more likely to respond. As a result, survey responses may not be generalizable. However, this survey has one of the largest number of respondents of GE fellows in the United States, both in overall quantity of fellows, variety of institutions they belong to, and in the number of trainee respondents who have had real hands-on exposure to CADe. In addition, less-experienced trainees may tend to be more overconfident and respond as such, according to the Dunning-Kruger effect, which could affect their responses.^20^ Future research should objectively assess the impact of AI on fellows' cognitive endoscopic skills to determine whether and when this technology should be integrated into fellowship training and to inform the development of optimized AI-based endoscopy curricula.

## Conclusion

Our nationwide survey found that 69% of GE fellows are already using CADe for colonoscopy in at least 1 training site. GE fellows are generally supportive of integrating CADe into their training, with many advocating for its incorporation in the second year of training. However, training programs should be developed through appropriate clinical research. Furthermore, those trained with AI preferred continued access to CADe when in independent practice. These should be considered by fellowship program leadership and GE practices recruiting fellows trained with AI.

## Data transparency statement

Data, analytic methods, and study materials will not be made available to other researchers.

## Patient consent

Consent was not required for this quality improvement study. Participation was completely voluntary.

## Disclosure

All authors disclosed no financial relationships.

## Figures and Tables

**Supplementary Table 1:** The remainder of the results of the survey not included in Table 1

Survey item	Available responses	% (n)
**Exposure and experience with AI-assisted colonoscopy in fellowship**		
Approximately how many outpatient colonoscopies have you performed using AI for polyp detection (CADe) technology?	Between 1 and 25 colonoscopies. Between 26 and 50 colonoscopies Between 51 and 75 colonoscopies. Between 76 and 100 colonoscopies. >100 colonoscopies.	21.7% (13) 25.0% (15) 20% (12) 11.7% (7) 21.7% (13)
My attendings generally allow me to decide when to use AI for polyp detection (CADe) during colonoscopy procedures.	Never. Less than half of the time. About half of the time. Most of the time. Always. Do not know. Prefer not to answer.	21.8% (12) 9.1% (5) 7.3% (4) 29.1% (16) 30.9% (17) 0.0% (0) 1.8% (1)
Which of the following has your fellowship program provided to teach you about the principles and application of AI-assisted colonoscopy? Check all that apply.	Lectures about AI-assisted colonoscopy. AI simulations or workshops. AI-related journal clubs. Other methods. None of the above. Do not know.	63.9% (23) 11.1% (4) 44.4% (16) 8.3% (3) 0.0% (0) 0.0% (0)
The attendings I have worked with are generally supportive/positive about the use of AI in colonoscopy for polyp detection (CADe).	Strongly agree. Somewhat agree. Neither agree nor disagree. Somewhat disagree. Strongly disagree.	36.7% (22) 45.0% (27) 15.0% (9) 3.3% (2) 0.0% (0)
Have you ever visualized a polyp during a colonoscopy that was not detected or captured effectively with AI for polyp detection (CADe)?	Yes. No. Do not know.	66.0% (35) 34.0% (18) 0.0% (0)
How often do you visualize polyps before AI captures them with a bounding box?	Never. Rarely. Often. Very often. Don't know.	1.7% (1) 32.2% (19) 45.8% (27) 8.5% (5) 11.9% (7)
**Attitudes toward AI-assisted colonoscopy**		
I believe that AI will be widespread in most or all endoscopic practices in the United States in the next 5 years.	Strongly agree. Somewhat agree. Neither agree nor disagree. Somewhat disagree. Strongly disagree.	46.0% (40) 42.5% (37) 11.5% (10) 0.0% (0) 0.0% (0)
I believe that AI leads to higher detection rates of adenomatous polyps than standard, unassisted colonoscopy.	Strongly agree. Somewhat agree. Neither agree nor disagree. Somewhat disagree. Strongly disagree.	29.9% (26) 42.5% (37) 20.7% (18) 6.9% (6) 0.0% (0)
I believe that AI decreases the chances of missing adenomas as compared with standard, unassisted colonoscopy.	Strongly agree. Somewhat agree. Neither agree nor disagree. Somewhat disagree. Strongly disagree.	31.8% (28) 56.8% (50) 10.2% (9) 0.0% (0) 1.1% (1)
I believe that AI decreases the chances of missing advanced adenomas compared with standard, unassisted colonoscopy.	Strongly agree. Somewhat agree. Neither agree nor disagree. Somewhat disagree. Strongly disagree.	25.0% (22) 28.4% (25) 21.6% (19) 20.5% (18) 4.5% (4)
I believe that AI decreases the chances of missing sessile serrated lesions compared with standard, unassisted colonoscopy.	Strongly agree. Somewhat agree. Neither agree nor disagree. Somewhat disagree. Strongly disagree.	29.5% (26) 50.0% (44) 18.2% (16) 1.1% (1) 1.1% (1)
I believe that AI leads to higher rates of removal of benign, nonadenomatous lesions.	Strongly agree. Somewhat agree. Neither agree nor disagree. Somewhat disagree. Strongly disagree.	41.4% (36) 28.7% (25) 21.8% (19) 8.0% (7) 0.0% (0)
I believe that AI makes colonoscopy withdrawal times longer.	Strongly agree. Somewhat agree. Neither agree nor disagree. Somewhat disagree. Strongly disagree.	26.1% (23) 34.1% (30) 30.7% (27) 8.0% (7) 1.1% (1)
Why do you think that AI-assisted colonoscopy for polyp detection makes withdrawal times longer?	AI finds more polyps, so the endoscopist spends more time removing them. It leads the endoscopist to do a more thorough mucosal evaluation. The endoscopist spends more time double-checking potential false-positive boxes. Do not know. Other.	45.3% (24) 41.5% (22) 92.5% (49) 0.0% (0) 0.0% (0)
I believe that hands-on exposure to AI should be available in all gastroenterology fellowship programs.	Strongly agree. Somewhat agree. Neither agree nor disagree. Somewhat disagree. Strongly disagree.	50.0% (44) 31.8% (28) 14.8% (13) 3.4% (3) 0.0% (0)
I believe that being exposed to AI early on in my endoscopic training will help me learn how to identify colorectal neoplasia.	Strongly agree. Somewhat agree. Neither agree nor disagree. Somewhat disagree. Strongly disagree.	26.2% (16) 34.4% (21) 19.7% (12) 13.1% (8) 6.6% (4)
I believe that if I were trained with AI, then I would miss some polyps if AI were not available to me in the future.	Strongly agree. Somewhat agree. Neither agree nor disagree. Somewhat disagree. Strongly disagree.	17.0% (15) 40.9% (36) 23.9% (21) 14.8% (13) 3.4% (3)
I believe that the use of AI has made me a better endoscopist.	Strongly agree. Somewhat agree. Neither agree nor disagree. Somewhat disagree. Strongly disagree.	13.1% (8) 18.0% (11) 52.5% (32) 9.8% (6) 6.6% (4)
I am interested in adopting AI as part of my endoscopic training during fellowship and beyond into my career.	Strongly agree. Somewhat agree. Neither agree nor disagree. Somewhat disagree. Strongly disagree.	40.9% (36) 40.9% (36) 11.4% (10) 6.8% (6) 0.0% (0)
If I were trained with AI, I would prefer to pursue a job with this technology available after the conclusion of my training.	Strongly agree. Somewhat agree. Neither agree nor disagree. Somewhat disagree. Strongly disagree.	29.5% (26) 33.0% (29) 31.8% (28) 4.5% (4) 1.1% (1)

*AI*, Artificial intelligence; *CADe*, computer-aided detection.

## Supplementary data

Supplementary Figure 1 The entirety of the 29-question survey.
