# Supplementary material for: Training future endoscopists: gastroenterology fellows' perspectives and hands-on exposure to artificial intelligence for polyp detection in the United States
Source: IGIE. 2026 Feb 2;5(2):167–172.e2. doi: 10.1016/j.igie.2026.01.016 (PMC13324113; doi:10.1016/j.igie.2026.01.016)
Supplement: Supplementary Figure 1 — The entirety of the 29-question survey. [file mmc1.pdf]

Trainees' Perspectives and Exposure to Artificial Intelligence: A Survey for GI Fellows

Start of Block: Exposure and Experience to Computer-Aided Detection (CADe) in Fellowship

Q1 Please indicate which types of locations you rotate through as part of your fellowship training. *Check all that apply.*

- ▢ University hospital (1)
- ▢ Community hospital (2)
- ▢ VA medical center (3)
- ▢ Ambulatory surgery center (4)
- ▢ Other (please specify) (5) ____

Q2 Is artificial intelligence-assisted colonoscopy for polyp detection (CADe) available at least at one of your training sites?

- ○ Yes, AI is available for fellows to use. (1)
- ○ Yes, AI is available, but not for fellows to use. (2)
- ○ No, AI is not available. (3)

Display this question:

If Please indicate which types of locations you rotate through as part of your fellowship training.... = University hospital

And Is artificial intelligence-assisted colonoscopy for polyp detection (CADe) available at least at... != No, AI is not available.

Q2a Is AI for polyp detection (CADe) available during colonoscopy at the university hospital?

- ◯ Yes, AI is available for fellows to use. (1)
- ◯ Yes, AI is available, but not for fellows to use. (2)
- ◯ No, AI is not available. (3)

Display this question:

If Please indicate which types of locations you rotate through as part of your fellowship training.... = Community hospital

And Is artificial intelligence-assisted colonoscopy for polyp detection (CADe) available at least at... != No, AI is not available.

Q2b Is AI for polyp detection (CADe) available during colonoscopy at the community hospital?

- ◯ Yes, AI is available for fellows to use. (1)
- ◯ Yes, AI is available, but not for fellows to use. (2)
- ◯ No, AI is not available. (3)

Display this question:

If Please indicate which types of locations you rotate through as part of your fellowship training.... = VA medical center

And Is artificial intelligence-assisted colonoscopy for polyp detection (CADe) available at least at... != No, AI is not available.

Q2c Is AI for polyp detection (CADe) available during colonoscopy at the VA medical center?

◯ Yes, AI is available for fellows to use. (1)

◯ Yes, AI is available, but not for fellows to use. (2)

◯ No, AI is not available. (3)

Display this question:

If Please indicate which types of locations you rotate through as part of your fellowship training.... = Ambulatory surgery center

And Is artificial intelligence-assisted colonoscopy for polyp detection (CADe) available at least at... != No, AI is not available.

Q2d Is AI for polyp detection (CADe) available during colonoscopy at the ambulatory surgery center?

◯ Yes, AI is available for fellows to use. (1)

◯ Yes, AI is available, but not for fellows to use. (2)

◯ No, AI is not available. (3)

Display this question:

If Please indicate which types of locations you rotate through as part of your fellowship training.... = Other (please specify)

And Is artificial intelligence-assisted colonoscopy for polyp detection (CADe) available at least at... != No, AI is not available.

Q2e Is AI for polyp detection (CADe) available during colonoscopy at the other center you rotate at?

◯ Yes, AI is available for fellows to use. (1)

◯ Yes, AI is available, but not for fellows to use. (2)

◯ No, AI is not available. (3)

Display this question:

If Is AI for polyp detection (CADe) available during colonoscopy at the university hospital? = Yes, AI is available for fellows to use.

Q3a Are there any limitations to the use of AI for polyp detection (CADe) for trainees at the university hospital?

- No limitations. (1)
- Use is limited to 3rd year fellows. (2)
- Use is limited to 2nd year fellows and above. (3)
- Use is limited to attendings only. (4)

Display this question:

If Is AI for polyp detection (CADe) available during colonoscopy at the community hospital? = Yes, AI is available for fellows to use.

Q3b Are there any limitations to the use of AI for polyp detection (CADe) for trainees at the community hospital?

- No limitations. (1)
- Use is limited to 3rd year fellows. (2)
- Use is limited to 2nd year fellows and above. (3)
- Use is limited to attendings only. (4)

Display this question:

If Is AI for polyp detection (CADe) available during colonoscopy at the VA medical center? = Yes, AI is available for fellows to use.

Q3c Are there any limitations to the use of AI for polyp detection (CADe) for trainees at the VA medical center?

- No limitations. (1)
- Use is limited to 3rd year fellows. (2)
- Use is limited to 2nd year fellows and above. (3)
- Use is limited to attendings only. (4)

Display this question:

If Is AI for polyp detection (CADe) available during colonoscopy at the ambulatory surgery center? = Yes, AI is available for fellows to use.

Q3d Are there any limitations to the use of AI for polyp detection (CADe) for trainees at the ambulatory surgery center?

- No limitations. (1)
- Use is limited to 3rd year fellows. (2)
- Use is limited to 2nd year fellows and above. (3)
- Use is limited to attendings only. (4)

Display this question:

If Is AI for polyp detection (CADe) available during colonoscopy at the other center you rotate at? = Yes, AI is available for fellows to use.

Q3e Are there any limitations to the use of AI for polyp detection (CADe) for trainees at the other site you rotate at?

- ◯ No limitations. (1)
- ◯ Use is limited to 3rd year fellows. (2)
- ◯ Use is limited to 2nd year fellows and above. (3)
- ◯ Use is limited to attendings only. (4)

Q4 Approximately how many colonoscopies have you performed, with or without AI for polyp detection (CADe)?

- ◯ Please enter here (1) ____

Display this question:

If Is artificial intelligence-assisted colonoscopy for polyp detection (CADe) available at least at... = Yes, AI is available for fellows to use.

Q5 Approximately how many outpatient colonoscopies have you performed using AI for polyp detection (CADe) technology?

- ◯ Between 1-25 colonoscopies. (1)
- ◯ Between 26-50 colonoscopies. (2)
- ◯ Between 51-75 colonoscopies. (3)
- ◯ Between 76-100 colonoscopies. (4)
- ◯ Greater than 100 colonoscopies. (5)

Display this question:

If Is artificial intelligence-assisted colonoscopy for polyp detection (CADe) available at least at... = Yes, AI is available for fellows to use.

Q6 My attendings generally allow me to decide when to use AI for polyp detection (CADe) during colonoscopy procedures.

- Never (1)
- Less than half of the time (2)
- About half of the time (3)
- Most of the time (4)
- Always (5)
- Don't know (6)
- Prefer not to answer (7)

Q7 Which of the following has your fellowship program provided to teach you about the principles and application of AI-assisted colonoscopy? *Check all that apply.*

- Lectures about AI-assisted colonoscopy (1)
- AI simulations or workshops (2)
- AI-related journal clubs (3)
- Other methods: please specify (4) ____
- None of the above (5)
- Don't know (6)

Display this question:

If Is artificial intelligence-assisted colonoscopy for polyp detection (CADe) available at least at... != No, AI is not available.

Q8 The attendings I have worked with are generally supportive/positive about the use of AI in colonoscopy for polyp detection (CADe).

- ○ Strongly agree (1)
- ○ Somewhat agree (2)
- ○ Neither agree nor disagree (3)
- ○ Somewhat disagree (4)
- ○ Strongly disagree (5)

Display this question:

If Is artificial intelligence-assisted colonoscopy for polyp detection (CADe) available at least at... = Yes, AI is available for fellows to use.

Q9 Have you ever visualized a polyp during a colonoscopy that was not detected or captured effectively with AI for polyp detection (CADe)?

- ○ Yes (1)
- ○ No (2)
- ○ Don't know (3)

Display this question:

If Is artificial intelligence-assisted colonoscopy for polyp detection (CADe) available at least at... = Yes, AI is available for fellows to use.

Q10 How often do you visualize polyps before AI captures them with a bounding box?

- Never (1)
- Rarely (2)
- Often (3)
- Very often (4)
- Don't know (5)

End of Block: Exposure and Experience to Computer-Aided Detection (CADe) in Fellowship

---

Start of Block: Attitudes Towards AI-Assisted Colonoscopy

Q11 I believe that AI-assisted colonoscopy for polyp detection (CADe) will be widespread in most or all endoscopic practices in the United States in the next 5 years.

- Strongly agree (1)
- Somewhat agree (2)
- Neither agree nor disagree (3)
- Somewhat disagree (4)
- Strongly disagree (5)

Q12 I believe that AI-assisted colonoscopy for polyp detection (CADe) leads to higher detection rates of adenomatous polyps compared to standard, unassisted colonoscopy.

- ○ Strongly agree (1)
- ○ Somewhat agree (2)
- ○ Neither agree nor disagree (3)
- ○ Somewhat disagree (4)
- ○ Strongly disagree (5)

Q13 I believe that AI-assisted colonoscopy for polyp detection (CADe) decreases the chances of missing adenomas as compared to standard, unassisted colonoscopy.

- ○ Strongly agree (1)
- ○ Somewhat agree (2)
- ○ Neither agree nor disagree (3)
- ○ Somewhat disagree (4)
- ○ Strongly disagree (5)

Q14 I believe that AI-assisted colonoscopy for polyp detection (CADe) decreases the chances of missing advanced adenomas (high-grade dysplasia, contains villous histology, and/or is 10 mm or larger in size) compared to standard, unassisted colonoscopy.

- ◯ Strongly agree (1)
- ◯ Somewhat agree (2)
- ◯ Neither agree nor disagree (3)
- ◯ Somewhat disagree (4)
- ◯ Strongly disagree (5)

Q15 I believe that AI-assisted colonoscopy for polyp detection (CADe) decreases the chances of missing sessile serrated lesions compared to standard, unassisted colonoscopy.

- ◯ Strongly agree (1)
- ◯ Somewhat agree (2)
- ◯ Neither agree nor disagree (3)
- ◯ Somewhat disagree (4)
- ◯ Strongly disagree (5)

Q16 I believe that AI-assisted colonoscopy for polyp detection (CADe) leads to higher rates of removal of benign, non-adenomatous lesions.

- ◯ Strongly agree (1)
- ◯ Somewhat agree (2)
- ◯ Neither agree nor disagree (3)
- ◯ Somewhat disagree (4)
- ◯ Strongly disagree (5)

Q17 I believe that AI-assisted colonoscopy for polyp detection (CADe) makes colonoscopy withdrawal times longer.

- ◯ Strongly agree (1)
- ◯ Somewhat agree (2)
- ◯ Neither agree nor disagree (3)
- ◯ Somewhat disagree (4)
- ◯ Strongly disagree (5)

Display this question:

If I believe that AI-assisted colonoscopy for polyp detection (CADe) makes colonoscopy withdrawal ti... = Strongly agree

Or I believe that AI-assisted colonoscopy for polyp detection (CADe) makes colonoscopy withdrawal ti... = Somewhat agree

Q17a Why do you think that AI-assisted colonoscopy for polyp detection (CADe) makes withdrawal times longer? *Check all that apply.*

▢ AI finds more polyps, so the endoscopist spends more time removing them. (6)

▢ It leads the endoscopist to do a more thorough mucosal evaluation. (7)

▢ The endoscopist spends more time double-checking potential false positive boxes. (9)

▢ Don't know (10)

▢ Other (11) ____

Q18 I believe that hands-on exposure to AI-assisted colonoscopy for polyp detection (CADe) should be available in all gastroenterology fellowship programs.

○ Strongly agree (1)

○ Somewhat agree (2)

○ Neither agree nor disagree (3)

○ Somewhat disagree (4)

○ Strongly disagree (5)

Display this question:

If Is artificial intelligence-assisted colonoscopy for polyp detection (CADe) available at least at... = Yes, AI is available for fellows to use.

Q19 I believe that being exposed to AI-assisted colonoscopy for polyp detection (CADe) early on in my endoscopic training will help me learn how to identify colorectal neoplasia.

- Strongly agree (1)
- Somewhat agree (2)
- Neither agree nor disagree (3)
- Somewhat disagree (4)
- Strongly disagree (5)

Q20 I believe that if I was trained with AI-assisted colonoscopy for polyp detection (CADe), then I would miss some polyps if AI was not available to me in the future.

- Strongly agree (1)
- Somewhat agree (2)
- Neither agree nor disagree (3)
- Somewhat disagree (4)
- Strongly disagree (5)

Q21 When do you think would be the ideal timing to implement training in the use of AI-assisted colonoscopy for polyp detection (CADe) into endoscopic training?

- 1st year of fellowship (1)
- 2nd year of fellowship (2)
- 3rd year of fellowship (3)
- I don't think it should be incorporated into fellowship training. (4)

Display this question:

If Is artificial intelligence-assisted colonoscopy for polyp detection (CADe) available at least at... = Yes, AI is available for fellows to use.

Q22 I believe that the use of AI-assisted colonoscopy for polyp detection (CADe) has made me a better endoscopist.

- Strongly agree (1)
- Somewhat agree (2)
- Neither agree nor disagree (3)
- Somewhat disagree (4)
- Strongly disagree (5)

Q23 I am interested in adopting AI-assisted colonoscopy for polyp detection (CADe) as part of my endoscopic training during fellowship and beyond into my career.

- Strongly agree (1)
- Somewhat agree (2)
- Neither agree nor disagree (3)
- Somewhat disagree (4)
- Strongly disagree (5)

Q24 If I was trained with AI-assisted colonoscopy for polyp detection (CADe), I would prefer to pursue a job with this technology available after conclusion of my training.

- ○ Strongly agree (1)
- ○ Somewhat agree (2)
- ○ Neither agree nor disagree (3)
- ○ Somewhat disagree (4)
- ○ Strongly disagree (5)

End of Block: Attitudes Towards AI-Assisted Colonoscopy

Start of Block: Demographic Information

Q25 What is your gender?

- ○ Male (1)
- ○ Female (2)
- ○ Non-binary (3)
- ○ Transgender male (4)
- ○ Transgender female (5)
- ○ Prefer not to answer (6)

Q26 Which gastroenterology fellowship do you attend? (This information will help us continue to follow-up with you regarding the impact of AI in your training. If you prefer not to answer, you can write that.)

- ○ Enter your fellowship program here (1) ____

Q27 What is your current year of training in gastroenterology fellowship?

- ☐ 1st year fellow (1)
- ☐ 2nd year fellow (2)
- ☐ 3rd year fellow (3)
- ☐ Advanced fellow (e.g. IBD, advanced endoscopy, transplant hepatology) (4)
- ☐ Prefer not to answer (5)

Q28 Which of the following best describes your plans after fellowship training?

- ○ General gastroenterology (1)
- ○ Hepatology (2)
- ○ IBD focus (3)
- ○ Advanced Endoscopy (4)
- ○ To be determined (5)

Q29 If interested, please provide a personal email address for a future survey once you transition out of fellowship. We want to better understand the impact of having AI available during training to see how it affects your future practice.

- ○ Enter personal email address here (1) ____

End of Block: Demographic Information
